# Genetics of kidney disease and related cardiometabolic phenotypes in Zuni Indians: the Zuni Kidney Project

**DOI:** 10.3389/fgene.2015.00006

**Published:** 2015-01-30

**Authors:** Sandra L. Laston, V. Saroja Voruganti, Karin Haack, Vallabh O. Shah, Arlene Bobelu, Jeanette Bobelu, Donica Ghahate, Antonia M. Harford, Susan S. Paine, Francesca Tentori, Shelley A. Cole, Jean W. MacCluer, Anthony G. Comuzzie, Philip G. Zager

**Affiliations:** ^1^South Texas Diabetes and Obesity Institute, Regional Academic Health Center, University of Texas at San AntonioHarlingen, TX, USA; ^2^Department of Nutrition, University of North Carolina at Chapel HillKannapolis, NC, USA; ^3^University of North Carolina Nutrition Research Institute, University of North Carolina at Chapel HillKannapolis, NC, USA; ^4^Department of Genetics, Texas Biomedical Research InstituteSan Antonio, TX, USA; ^5^Department of Biochemistry, University of New Mexico School of MedicineAlbuquerque, NM, USA; ^6^Dialysis Clinic, Inc., Albuquerque, NMUSA; ^7^Arbor Research Collaborative for HealthAnn Arbor, MI, USA; ^8^Southwest National Primate Research CenterSan Antonio, TX, USA; ^9^Department of Medicine, Division of Nephrology, University of New Mexico School of MedicineAlbuquerque, NM, USA

**Keywords:** single nucleotide polymorphisms, association, kidney function, serum uric acid, triglycerides

## Abstract

The objective of this study is to identify genetic factors associated with chronic kidney disease (CKD) and related cardiometabolic phenotypes among participants of the Genetics of Kidney Disease in Zuni Indians study. The study was conducted as a community-based participatory research project in the Zuni Indians, a small endogamous tribe in rural New Mexico. We recruited 998 members from 28 extended multigenerational families, ascertained through probands with CKD who had at least one sibling with CKD. We used the Illumina Infinium Human1M-Duo version 3.0 BeadChips to type 1.1 million single nucleotide polymorphisms (SNPs). Prevalence estimates for CKD, hyperuricemia, diabetes, and hypertension were 24%, 30%, 17% and 34%, respectively. We found a significant (*p* < 1.58 × 10^-7^) association for a SNP in a novel gene for serum creatinine (*PTPLAD2*). We replicated significant associations for genes with serum uric acid (*SLC2A9*), triglyceride levels (*APOA1*, *BUD13*, *ZNF259*), and total cholesterol (*PVRL2*). We found novel suggestive associations (*p* < 1.58 × 10^-6^) for SNPs in genes with systolic (*OLFML2B*), and diastolic blood pressure (*NFIA*). We identified a series of genes associated with CKD and related cardiometabolic phenotypes among Zuni Indians, a population with a high prevalence of kidney disease. Illuminating genetic variations that modulate the risk for these disorders may ultimately provide a basis for novel preventive strategies and therapeutic interventions.

## INTRODUCTION

The Zuni Indians are experiencing interrelated epidemics of chronic kidney disease (CKD) and related features of the cardiometabolic syndrome including obesity, diabetes, dyslipidemia, and hypertension that are intermediate phenotypes for CKD (Stidley et al., 2002; Shah et al., 2003; Scavini et al., 2007; MacCluer et al., 2010). Ethnicity also influences the risk for the development of CKD and related phenotypes (Johnson et al., 2009). Genetic studies, including candidate gene and genome-wide association studies (GWAS), have been conducted to elucidate the effects of specific genes on the variation in CKD and cardiometabolic risk factors. These include studies conducted in Caucasians (Hwang et al., 2007; Parsa et al., 2013), African Americans (Edwards et al., 2008; Willer et al., 2013; Bidulescu et al., 2014), Asians (Yamakawa-Kobayashi et al., 2012; Willer et al., 2013), Mexican Americans (Farook et al., 2013; Thameem et al., 2013), Pima Indians (Bian et al., 2013; Hanson et al., 2013, 2014), and in the 13 American Indian tribes participating in the Strong Heart Family Study (Franceschini et al., 2013; Voruganti et al., 2014). To decrease the burden of kidney disease and related intermediate phenotypes in the Zuni Pueblo, we established the Zuni Kidney Project (ZKP) in partnership with the Indian Health Service, University of New Mexico Health Sciences Center, Texas Biomedical Research Institute and Dialysis Clinic, Inc. (DCI; Stidley et al., 2002).

The Zuni Indians reside in the Zuni Pueblo, located in McKinley County, NM, USA. The population was 6,302 in the 2010 US Census and 97% of inhabitants were American Indians (Zuni Pueblo Quick Facts). Emigration and immigration rates are low and therefore the population is relatively endogamous. The majority of adults work as artisans, making jewelry and fetishes. The ZKP previously conducted a population-based, cross-sectional survey that reported high prevalence estimates, age-and sex-adjusted to the Zuni population, for decreased estimated glomerular filtration rate (eGFR; Scavini et al., 2007), albuminuria (Shah et al., 2003), and hematuria (Tentori et al., 2003). Prevalence estimates for albuminuria and hematuria were higher for diabetic than non-diabetic participants (Shah et al., 2003). The prevalence of end-stage renal disease (ESRD) among the Zuni Indians, adjusted for age and gender, was 20.0-, 4.4-, and 5.6-fold higher than that among European– and African–Americans and the composite estimate for all American Indians (Shah et al., 2003).

Recently the ZKP conducted the Genetics of Kidney Disease in Zuni Indians (GKDZI) Study to explore the hypothesis that genetic factors modulate susceptibility to CKD and related phenotypes. Studies of extended families, such as GKDZI, offer advantages over studies of sib pairs or unrelated individuals for gene discovery since they have enhanced statistical power, are more homogenous and allow for greater genotyping quality control (Laird and Lange, 2008). The current manuscript presents the results of a GWAS in extended, multigenerational families of Zuni Indians.

## MATERIALS AND METHODS

### STUDY DESIGN

We conducted a GWAS in extended families of Zuni Indians. The study cohort consisted of 30 extended families, of which 28 were multigenerational. The families were ascertained through probands with kidney disease, who had at least one sibling with kidney disease. The Institutional Review Boards of all participating institutions and the Zuni Tribal Council approved the protocol. All participants gave written informed consent.

### SETTING

The study was conducted in the Zuni Pueblo. Recruitment occurred from February 2005 through May 2009. Data were collected from February 2005 through June 2009.

### PARTICIPANTS

**FIGURE 1:**
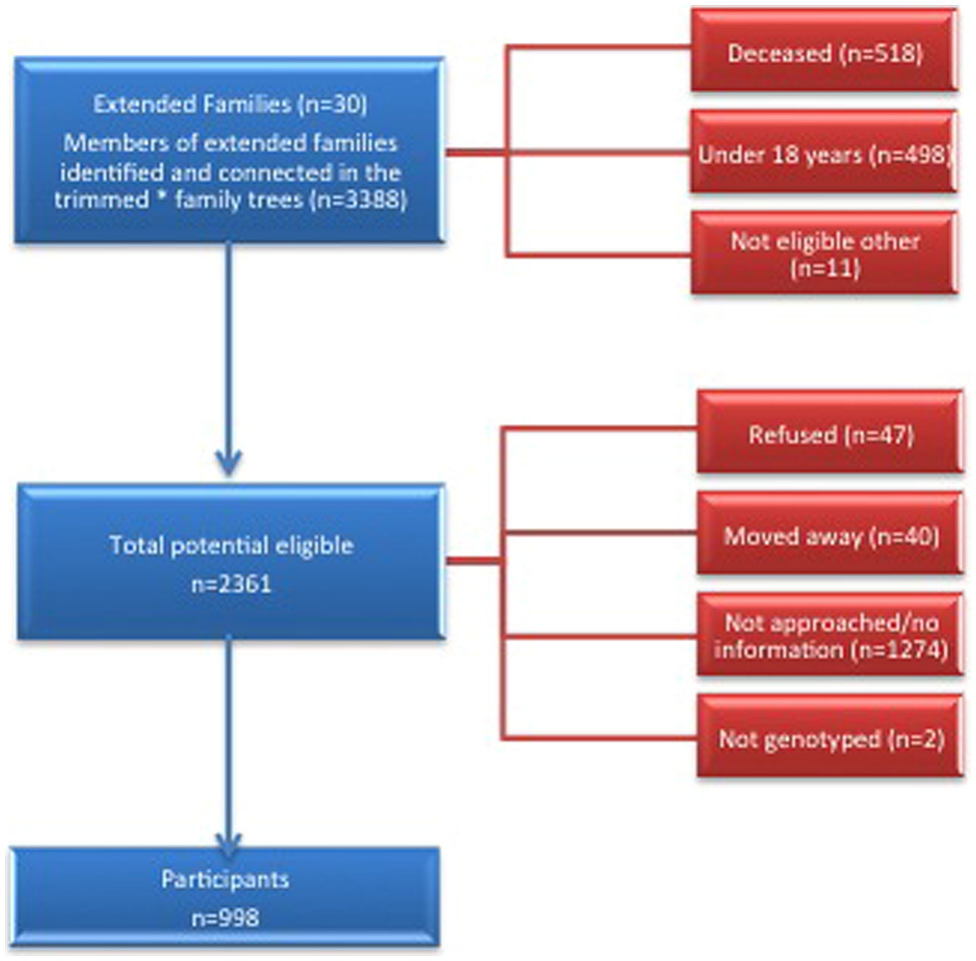
**Study participants, flow diagram**.

We conducted a cross-sectional study of extended families ascertained through probands with CKD who had at least one sibling with CKD. Potential probands were identified from the ZKP’s previous population-based study of kidney disease, which estimated the prevalence of incipient [15%, (13.1–16.9%)] and overt [4.7% (3.6–5.8%)] albuminuria among 1483 participants (Shah et al., 2003). Eligibility criteria for probands and affected siblings included age ≥18 years, a urine albumin to creatinine ratio (UACR) ≥0.2 in at least two of three urine samples or a reduced serum creatinine-based eGFR, modified for American Indians (Shara et al., 2012) using the Chronic Renal Insufficiency Cohort (CRIC) criteria (Feldman et al., 2003). We invited all first-degree (parents, siblings, and offspring), second-degree (aunts, uncles, nieces, nephews, grandparents, and grandchildren) and third-degree (first cousins, great aunts, great uncles, etc.) relatives of probands and their spouses over 18 years of age to participate. See the consort diagram for details of the recruitment process (**Figure 1**). We used PEDSYS for data entry, quality control, report generation, and preparation of data files for statistical genetic analysis (Dyke, 1994).

### PHENOTYPIC VARIABLES

A random blood sample was drawn from each participant. Blood was drawn for chemistry profile, hemoglobin A_1c_ (HbA_1c_), and serum creatinine and serum uric acid (SUA). We also measured serum triglycerides, HDL-, LDL-, and total serum cholesterol. We considered a participant to have diabetes if they met ≥1 of the following conditions: (1) history of diabetes, (2) random plasma glucose level ≥200 mg/dL (American Diabetes Association, 2012), (3) HbA_1c_ ≥6.5% (American Diabetes Association, 2012), (4) receiving diabetes medication(s). Three urine samples were collected on separate days from each participant. A participant was considered to have CKD if UACR ≥0.2 in ≥2 of 3 urine samples or if the eGFR was reduced. We also measured blood pressure and calculated body mass index (BMI). Participants were classified as hypertensive if they met ≥1 of the following conditions: (1) history of hypertension; (2) SBP or DBP ≥140 and ≥90 mm Hg, respectively (73–75), or (3) receiving antihypertensive medication(s).

### GENOTYPIC VARIABLES

DNA samples were obtained from peripheral blood mononuclear cells. We conducted genome-wide genotyping for 998 participants using the Illumina Human1M-Duo V3.0 BeadChips (Illumina, San Diego, CA, USA) that contain ~1.1 million single nucleotide polymorphism (SNP) assays. Illumina included sample-dependent and independent controls on their chips to test for accuracy of the procedure. Genotype calls were obtained after scanning on the Illumina BeadStation 500GX and analysis using the GenomeStudio software.

### GENOTYPING QUALITY CONTROL

Specific SNPs were removed from analysis if they had call rates <95% (4,867 SNPs), deviated from Hardy–Weinberg equilibrium (15), were mono-allelic (136,917) or had rare alleles occurring in fewer than five individuals (85,397). SNP genotypes were checked for Mendelian consistency using the program SimWalk (Sobel and Lange, 1996). The estimates of allele frequencies and their SE were obtained by a maximum likelihood estimation method that accounts for pedigree structure using the Sequential Oligogenic Linkage Analysis Routines (SOLAR; http://solar.txbiomedgenetics.org), version 4.3 (Almasy and Blangero, 1998), a program package that is used for association analysis, linkage analysis, transmission disequilibrium tests, and other statistical genetic analyses. Linkage disequilibrium, taking relatedness into account, was also calculated using SOLAR. Missing genotypes were imputed from pedigree data using MERLIN version 1.1.2 (Abecasis et al., 2002).

### REDUCING BIAS IN BIOLOGICAL SAMPLES

#### Reducing bias in UACR

To minimize classification bias, we obtained three urine samples from each participant. The median interval between urine collections was 2 days. We classified albuminuria and hematuria using the mode of three urine samples. UACR was classified as normal (<0.03), incipient (0.03–0.19), or overt (≥0.20). If all three samples were discordant, we used the median value. Urine albumin was measured using nephelometry (Liu et al., 2011; Nicol et al., 2011).

#### Reducing bias in eGFR

We used the four-variable Modification of Diet in Renal Disease (MDRD) Study equation, modified for use in American Indians to estimate GFR based on a single serum sample (Shah et al., 2003; Scavini et al., 2007). Limitations of this equation include limited validation data in American Indians and the lack of calibration of the serum creatinine assay. Serum creatinine levels are influenced by muscle mass. We recognize that the CKD-EPI equation may out-perform the MDRD equation among people with near normal kidney function (Levey et al., 2009). Unfortunately, however, there are few data on the performance of the CKD-EPI equation among American Indians. We categorized eGFR using the National Kidney Foundation’s (2002) Kidney Disease Outcomes Quality Initiative (KDOQI; K/DOQI guidelines) and the CRIC age-specific criteria (Feldman et al., 2003). Hyperuricemia was defined as SUA >6 mg/dl in women and SUA >7 mg/dl in men.

#### Genome-wide association analysis (GWA analysis)

Measured genotype analyses were performed using SOLAR version 4.3 (Almasy and Blangero, 1998). The number of SNPs included in the GWA analysis was 884,161. All phenotypes were transformed by inverse normalization to meet assumptions of normality. We obtained residuals using linear regression models adjusted for age, sex, their interactions and higher order terms. Our subjects were ascertained for CKD. To adjust for ascertainment bias, we took a conservative approach by computing likelihood for pedigrees incorporating the CKD phenotype as an additional covariate for kidney function phenotypes (eGFR, UACR, and serum creatinine; Farook et al., 2012). Additional covariates included hypertension and diabetic status. Individuals excluded from analysis included those taking diabetes medications for analysis of HbA1c, antihypertensive medications for analysis of SBP and DBP, and statins for analysis of lipid traits (triglycerides, total-, HDL-, and LDL-cholesterol).

Each SNP genotype was converted in MERLIN version 1.1.2 (Abecasis et al., 2002) to a covariate measure equal to 0, 1, or 2 copies of the minor allele (or, for missing genotypes, the weighted covariate based on imputation). These covariates were included in the variance-components mixed models for measured genotype analyses (Boerwinkle et al., 1986) versus null models that incorporated the random effect of kinship and fixed effects such as age, sex, their interaction and higher order terms. For the initial GWA screen, we tested each SNP covariate independently as a one degree of freedom likelihood ratio test. An adjusted alpha value for significance, using a Moskvina–Schmidt calculation (Moskvina and Schmidt, 2008) based on the effective number of independent SNPs given LD (*n* = 323,965 SNPs) in the Zuni families, provided the adjusted genome-wide significant and genome-wide suggestive thresholds of 1.58 × 10^-7^ and 1.58 × 10^-6^, respectively. We performed the quantitative transmission disequilibrium test (QTDT) to test for population stratification (Havill et al., 2005). The power calculations were implemented in SOLAR 4.3.

## RESULTS

### STUDY PARTICIPANTS

The descriptive characteristics of the GKDZI participants for the variables included in the GWAS are presented in **Table 1**. The mean age was 37.1 ± 13.6 years and 52% were males. Nearly 19% of the participants were diabetic, 34% were hypertensive, 30% had hyperuricemia, and 24% had CKD at the time of the GKDZI clinic exam. The GWAS included 998 individuals with available DNA samples. Genotype distributions of all significantly associated SNPs conformed to the Hardy–Weinberg equilibrium. Population stratification was not significant as per the QTDT and therefore did not confound our associations.

**Table 1:** Characteristics of traits related to kidney disease, diabetes, and CVD in GKDZI participants.

Phenotypes	Trait	*N*	% or mean (CI)*	Range
Age	Age (years)	1000**	37.1 (36.3, 38.0)	18.0–93.1
Sex	Men (%)	1000	51.8%	
Obesity	BMI (kg/m^2^)	1000	29.6 (29.1, 30.0)	16.8–64.7
Diabetes	HbA_1c_	1000	5.8 (5.7, 5.9)	3.8–14.0
	Diabetes (%)	999	18.5%	
Serum Lipids	Total cholesterol (mg/dL)	992	181.3 (179.2, 183.9)	71.0–400
	HDL-C (mg/dL)	947	50.2 (49.1, 51.2)	17.0–131.0
	LDL-C (mg/dL)	814	98.8 (96.7, 100.9)	17.0–323.0
	Triglycerides (mg/dL)	992	169.0 (161.1, 176.8)	11.0–2000.0
Blood Pressure	SBP (mm Hg)	1000	122.4 (121.4, 123.4)	81.3–198.7
	DBP (mm Hg)	1000	77.6 (76.9, 78.3)	47.3–132.0
	Hypertensive (%)	1000	33.5%	
Kidney Function	Kidney disease (%)	1000	23.5%	
	Dialysis (%)	1000	1.2%	
	Kidney transplant (%)	1000	0.1%	
	Serum albumin	998	4.29 (4.26, 4.32)	2.0–5.5
	Urine albumin	985	12.7 (9.3, 16.1)	0.08–613.0
	Serum cystatin C	915	0.86 (0.82, 0.90)	0.44–7.89
	eGFR_MDRD-AI_	998	115.4 (113.5, 117.2)	4.3–249.3
	Serum creatinine (mg/dL)	999	0.90 (0.85, 0.95)	0.3–11.2
	Urine creatinine	985	130.1 (125.0, 135.2)	3.0–460.5
	UACR	985	112.7 (80.2, 145.2)	1.0–9378.5

*Confidence interval.

**Total number of participants in GKDZI study was 1000 of which two were not included in the genome-wide association studies (GWAS).

*BMI, body mass index; HbA_1c_, glycosylated hemoglobin; HDL-C, high density lipoprotein cholesterol; LDL-C, low density lipoprotein cholesterol; SBP, systolic blood pressure; DBP, diastolic blood pressure; eGFR_MDRD-AI_, glomerular filtration rate estimated according to the Modification of Diet and Renal Disease modified for American Indians; UACR, urine albumin to creatinine ratio Diabetes, any one of the following: (1) history of diabetes; (2) random plasma glucose ≥200 mg/dL; (3) HbA1c >7.0%; (4) taking diabetes medication(s).*

*Hypertensive = (1) history of hypertension; (2) SBP >140 mm Hg, and/or DBP >90 mm Hg; (3) currently taking antihypertensive medications.*

*Kidney disease = (1) UACR >0.03 in two of three spot urine samples; (2) eGFR_MDRD-AI_ <70; (3) renal replacement therapy.*

### GENOME-WIDE ASSOCIATION ANALYSIS

#### Kidney traits

**FIGURE 2:**
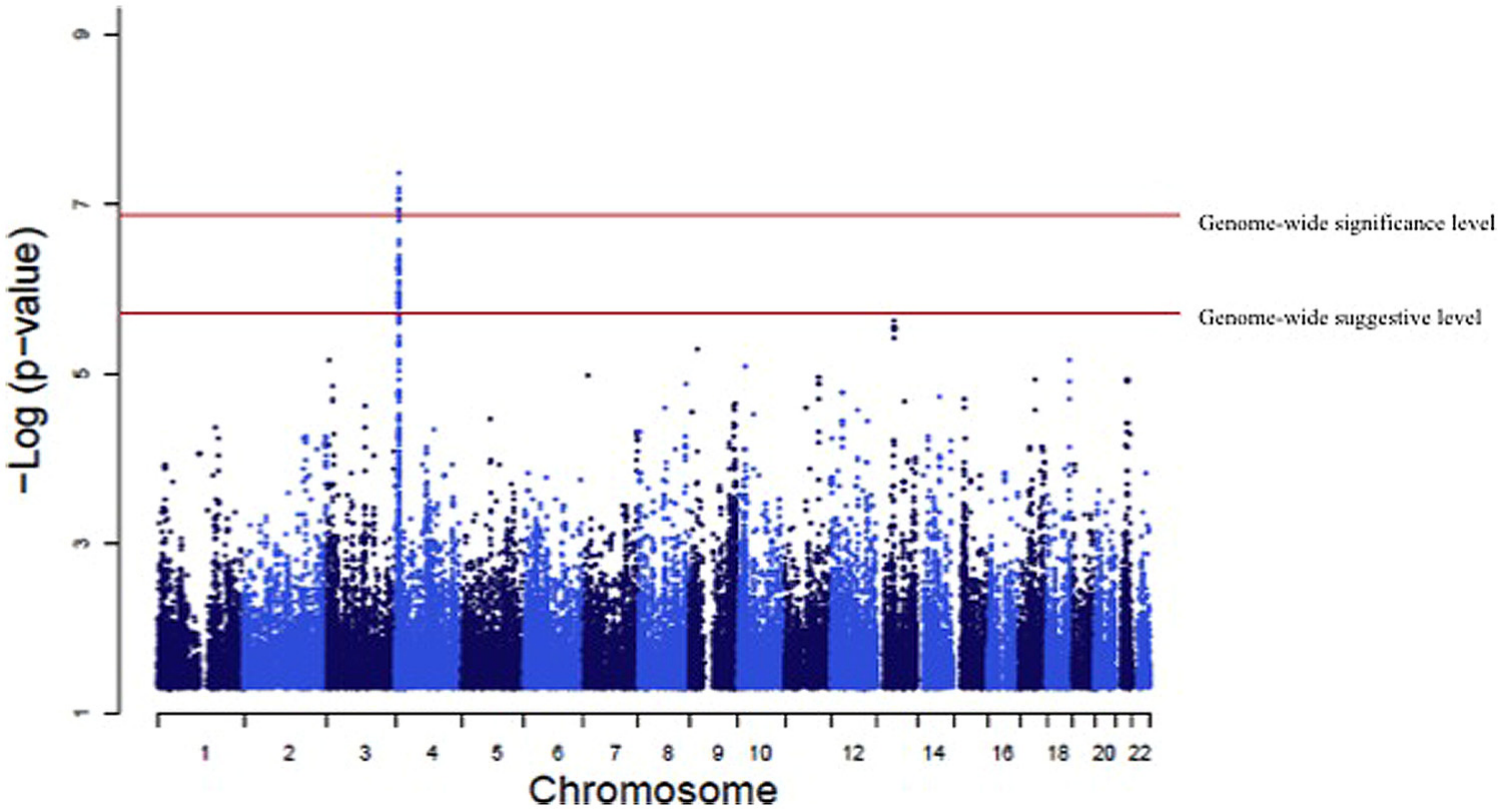
**Genome-wide association of serum uric acid (SUA) levels.** Manhattan Plot for the results of the genome wide association analysis with SUA levels. The genome-wide distribution of *p*-values for each of the SUA associated single nucleotide polymorphisms (SNPs) is shown. The adjusted genome-wide significant and genome-wide suggestive thresholds were set at 1.58 × 10^-7^ and 1.58 × 10^-6^, respectively. The x axis represents the genomic position of *SLC2A9* SNPs; the y axis shows the -log10 *p*-value. There were significant associations with 10 *SLC2A9* SNPs (rs6449213, rs938555, rs16890979, rs12499857, rs734553, rs6832439, rs13125646, rs13131257, rs13145758, and rs9998811).

A genome-wide significant association was identified for serum creatinine (**Table 2**). An intronic variant (minor allele G) in the protein tyrosine phosphatase-like A domain containing 2 (*PTPLAD2*) gene on Chromosome 9 was significantly associated (*p* = 1.2 × 10^-7^) with increased serum creatinine concentrations, with an effect size (residual phenotypic variance that is contributed by the minor allele of the SNP) of 3.0%. Evidence of suggestive association was found for serum creatinine with phospholipase A2 group 4a (*PLA2G4A*), ATPase, Class V, type 10B (*ATP10B*), and disks, large homolog 2 (Drosophila; *DLG2*; **Table 2**). However, we did not find any significant or suggestive associations for eGFR or the urine to albumin creatinine ratio (UACR). In addition, we found significant associations of SUA with several SNPs in solute carrier family 2, member 9 (*SLC2A9*) gene (rs6449213, rs938555, rs16890979, rs12499857, rs734553, rs6832439, rs13125646, rs13131257, rs13145758, and rs9998811; **Figure 2**). Minor alleles of most of these SNPs (shown in detail in **Table 3**) were associated with lower SUA levels.

**Table 2:** GWAS results for kidney function results in Zuni Indians.

Variable	N*	Chromosome	SNP	*p*-value for association	Major/minor allele	Minor allele frequency	Effect size	Gene symbol gb37	Gene name	Coordinate gb37	Gene location gb37
Serum albumin	981	16	rs8056272	1.05 × 10^-6^	A/C	0.33	0.02	*LOC100288121 LOC401859*	LOC401859: peptidyl-prolyl cis-trans isomerase A-like pseudogene (genecards)	73710475	INTERGENIC
Serum creatinine^2^	986	1	rs2383574	8.06 × 10^-7^	G/A	0.40	0.03	*PLA2G4A*	Phospholipase A2, group IVA	187081199	INTERGENIC
Serum creatinine	986	5	rs11135109	1.07 × 10^-6^	C/A	0.36	0.02	*ATP10B*	ATPase, class V, type 10B	160099440	INTRON
Serum creatinine	986	9	rs2275887	1.22 × 10^-7^	A/G	0.43	0.03	*PTPLAD2*	Protein tyrosine phosphatase-like A domain containing 2	21017828	gb37
Serum creatinine	986	11	rs17147179	9.00 × 10^-7^	G/A	0.06	0.03	*DLG2*	Disks, large homolog 2	84029748	INTRON

*^∗^number of samples included in GWA analysis.*

**Table 3:** Significant associations and genotype-specific means of serum uric acid (SUA) levels* (mg/dl).

*SLC2A9* SNP	Minor allele/frequency	*p*-value	Effect size^a^	A/A^c^	A/G^c^	G/G^c^
rs6449213	G/0.32	4.5 × 10^-8b^	4.1	6.14 (1.6)	5.80 (1.6)	5.21 (1.5)
rs16890979	G/0.49	6.4 × 10^-8^	3.7	5.53 (1.6)	5.90 (1.6)	6.22 (1.6)
rs938555	G/0.49	6.6 × 10^-8^	3.7	5.53 (1.6)	5.89 (1.6)	6.22 (1.6)
rs12499857	G/0.37	8.4 × 10^-8^	4.3	6.15 (1.6)	5.88 (1.6)	5.23 (1.5)
rs6832439	G/0.49	8.5 × 10^-8^	3.6	5.53 (1.6)	5.89 (1.6)	6.22 (1.6)
rs734553	A/0.49	8.5 × 10^-8^	3.6	6.24 (1.6)	5.88 (1.6)	5.56 (1.6)
rs13125646	G/0.49	1.1 × 10^-7^	3.5	5.56 (1.6)	5.88 (1.6)	6.24 (1.6)
rs13131257	G/0.49	1.1 × 10^-7^	3.5	5.56 (1.6)	5.88 (1.6)	6.24 (1.6)
rs13145758	A/0.49	1.1 × 10^-7^	3.5	6.24 (1.6)	5.88 (1.6)	5.56 (1.6)
rs9998811	G/0/49	1.1 × 10^-7^	3.5	5.56 (1.6)	5.88 (1.6)	6.24 (1.6)
rs7680126	G/0.49	1.3 × 10^-7^	3.5	6.21 (1.6)	5.88 (1.7)	5.54 (1.6)
rs881971	A/0.48	1.4 × 10^-7^	3.6	6.22 (1.6)	5.92 (1.6)	5.53 (1.6)
rs13111638	A/0.32	1.5 × 10^-7^	3.8	5.22 (1.5)	5.80 (1.6)	6.13 (1.6)

*^∗^number of samples included in GWAS analysis = 993.*

*^a^Proportion of residual phenotypic variance accounted by the minor allele of the single nucleotide polymorphism (SNP).*

*^b^Genome-wide significance level was set at *p* < 1.58 × 10^-7^.*

*^c^Mean (SD).*

#### Lipid phenotypes

**FIGURE 3:**
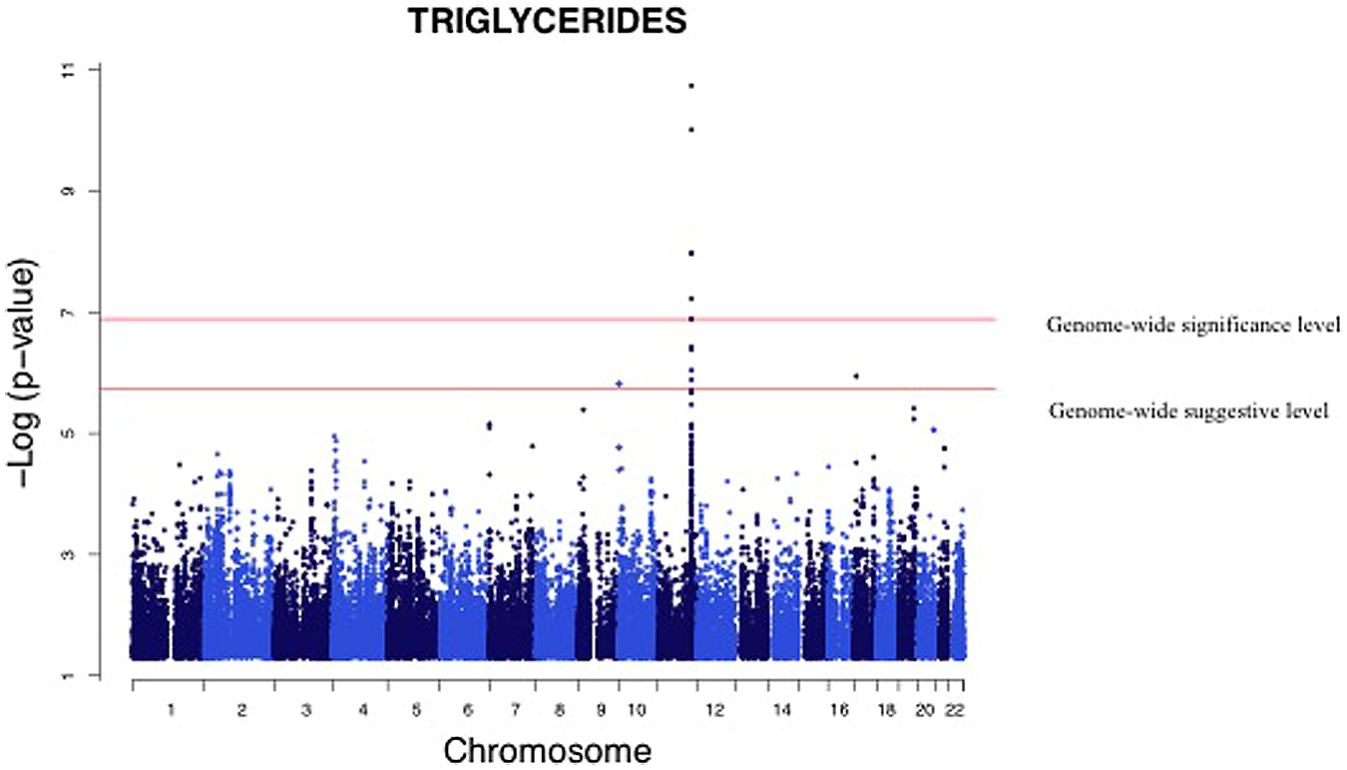
**Genome-wide association analysis of serum triglycerides.** Manhattan Plot for the results of the genome wide association analysis with serum triglyceride levels. The genome-wide distribution of *p*-values for each of the serum triglyceride associated SNPs is shown. The adjusted genome-wide significant and genome-wide suggestive thresholds were set at 1.58 × 10^-7^ and 1.58 × 10^-6^, respectively. The x axis represents the genomic position of the triglyceride associated SNPs; the y axis shows the -log10 *p*-value. The strongest association was for SNPs near the zinc finger protein 259 (*ZNF259*), apolipoprotein A-1 (*APOA1*), and BUD 13 homolog (*BUD13*) genes on Chromosome 11.

We analyzed the levels of four lipid phenotypes, e.g., triglycerides, high-density lipoprotein (HDL) cholesterol, low-density lipoprotein (LDL) cholesterol, and total cholesterol in the GWAS. The strongest association was found for triglycerides for SNPs near the zinc finger protein 259 (*ZNF259*), apolipoprotein A-1 (*APOA1*), and BUD13 homolog (*BUD13*) genes on Chromosome 11 (**Table 4**). Triglycerides were significantly associated (*p* = 1.83 × 10^-11^ to 6.00 × 10^-8^) with four SNPs near genes and one intronic SNP in *BUD13* whose mean effect size ranged from 3.2 to 4.4% (**Table 4**). All minor alleles of SNPs (effect sizes ranging between 2.3 and 4.8%) except rs180360 (effect size = 4.9%) were associated with increased triglycerides. Two of the SNPs near *BUD13* (rs10466588, rs6589563) were in complete LD. Two associated SNPs near the *APOA1* gene were also in complete LD. The mean effect size for the two SNPs was 3.6%. **Figure 3** provides a Manhattan Plot for the results of the genome-wide association analysis with triglyceride levels. The minor allele (C) of an intronic SNP (rs3852861) in the poliovirus receptor-related 2 (*PVRL2*) gene on Chromosome 19 was significantly associated (*p* = 6.44 × 10^-8^) with increased total cholesterol. The mean effect size was 3.4% (**Table 4**). We also found evidence of suggestive associations for triglycerides, HDL-, LDL-, and total cholesterol on Chromosomes 17, 16, 2, and 2, respectively.

**Table 4:** GWAS results for lipid traits in Zuni Indians.

Variable name	Chr	N*	SNP	*p*-value for association	Major/minor allele	Minor allele frequency	Effect size	Gene symbol gb37	Gene name	Coordinate gb37	Gene location gb37
Cholesterol	2	939	rs2666306	4.54 × 10^-7^	T/A	0.04	0.03	*MYADML*	Myeloid-associated differentiation marker-like	34069629	INTERGENIC
Cholesterol	19	939	rs3852861	6.44 × 10^-8^	A/C	0.18	0.03	*PVRL2*	Poliovirus receptor-related 2 (herpesvirus entry mediator B)	45383061	INTRON
HDL	16	897	rs7499892	1.09 × 10^-6^	G/A	0.15	0.03	*CETP*	Cholesteryl ester transfer protein, plasma	57006590	INTRON
LDL	2	775	rs12464255	3.93 × 10^-7^	G/A	0.13	0.04	*PLEKHM3*	Pleckstrin homology domain containing, family M, member 3	208926542	INTERGENIC
								*CRYGD*	Crystallin, gamma D		
Triglycerides	11	936	rs964184	1.83 × 10^-11^	G/C	0.39	0.05	*ZNF259*	Zinc finger protein 25	116648917	INTERGENIC
Triglycerides	11	936	rs180360	9.83 × 10^-11^	A/G	0.38	0.05	*BUD13*	BUD13 homolog	116598988	INTERGENIC
Triglycerides	11	936	rs6589563	1.04 × 10^-8^	G/A	0.44	0.04	*BUD13*	BUD13 homolog	116590787	INTERGENIC
Triglycerides	11	936	rs10466588	1.09 × 10^-8^	A/G	0.44	0.04	*BUD13*	BUD13 homolog	116610249	INTERGENIC
Triglycerides	11	936	rs180326	6.00 × 10^-8^	A/C	0.44	0.03	*BUD13*	BUD13 homolog	116624703	INTRON
Triglycerides	11	936	rs5072	1.29 × 10^-7^	G/A	0.31	0.04	*APOA1*	Apolipoprotein A-I	116707583	INTERGENIC
Triglycerides	11	936	rs2070665	1.29 × 10^-7^	G/A	0.31	0.04	*APOA1*	Apolipoprotein A-I	116707684	INTERGENIC
Triglycerides	11	936	rs541407	3.75 × 10^-7^	A/G	0.38	0.03	*BUD13*	BUD13 homolog	116313753	INTERGENIC
Triglycerides	11	936	rs11216129	4.16 × 10^-7^	C/A	0.24	0.03	*BUD13*	BUD13 homolog	116620256	INTRON
Triglycerides	11	936	rs1942478	9.15 × 10^-7^	A/C	0.23	0.03	*ZNF259*	Zinc finger protein 25	116651463	INTRON
Triglycerides	11	936	rs12272004	1.30 × 10^-6^	C/A	0.27	0.02	*BUD13*	BUD13 homolog	116603724	INTERGENIC
Triglycerides	17	936	rs2074258	1.14 × 10^-6^	G/A	0.22	0.03	*PIK3R6*	PIK3R6 phosphoinositide-3-kinase, regulatory subunit 6	8726160	INTRON

*Number of samples included in GWA analysis.

#### Blood pressure

Although GWA analysis of systolic (SBP) and diastolic blood pressure (DBP) yielded no significant associations, several exhibited evidence of suggestive associations (Data not shown). There were associations with two SNPs near the olfactomedin-like 2B (*OLFML2B*) gene on Chromosome 1 that approached significance (*p* = 9.68 × 10^-7^). The average effect size was 3.6% and was associated with increases in SBP. There was one intronic SNP in nuclear factor I/A (*NFIA*) on Chromosome 1 that showed evidence of suggestive association (*p* = 1.23 × 10^-6^) with decreased DBP.

## DISCUSSION

The most significant findings of the first GWAS in Zuni Indians were the strong associations of *PTPLAD2, SLC2A9*, *PVRL2,* and *BUD13* with serum levels of creatinine, uric acid, total cholesterol and triglycerides, respectively. Although, GWA analysis of BMI, SBP, DBP, and HbA1c provided no significant associations, some traits approached significance and several exhibited evidence of suggestive association.

We identified a novel significant association of an intronic variant in the *PTPLAD2* gene on Chromosome 9 with an increased serum creatinine concentration. This gene is part of very long chain fatty acid dehydratase HACD family and has a key role in the dehydration step of the very long chain fatty acid metabolism (Ikeda et al., 2008). Also implicated in tumor suppression (Zuni Pueblo QuickFacts from the US Census Bureau, 2014), this gene has not been previously reported to be associated with serum creatinine. We also found association, albeit suggestive, between serum creatinine and *PLA2G4A, ATP10B,* and *DLG2* SNPs. Their role in kidney function is not clear, except that in the kidney, cytosolic phospholipase A2 seems to play a role in GFR, vascular tone and water transport (Downey et al., 2001).

The strong association between SUA levels and *SLC2A9* SNPs is a replication and confirmation of these associations in several populations. Most of these studies were conducted in European populations (Doring et al., 2008; Li et al., 2008; Vitart et al., 2008) as well as in Asian (Tabara et al., 2010; Guan et al., 2011), African American (Dehghan et al., 2008; Rule et al., 2011; Tin et al., 2011) and Mexican American populations (Voruganti et al., 2013). The effect sizes or the proportion of residual variance in a phenotype that is explained by the minor allele of the SNP ranged between 3.5 and 4.3% in this study which is similar to what has been reported by these studies. Similar results were found in a candidate gene study in American Indians where only seven *SLC2A9* SNPs were genotyped (Voruganti et al., 2014). In addition, Caulfield et al. (2008) not only confirmed this association in six different cohorts of European ancestry but showed that SLC2A9 can exchange glucose for urate in the process of secretion of urate into the urine in functional studies.

Hyperuricemia is associated with hypertension (Johnson et al., 2005), CKD (Kim et al., 2014), insulin resistance (Cirillo et al., 2006), and cardiovascular disease (Puddu et al., 2012), although causality has not been established. SLC2A9 was originally identified as glucose transporter 9 (GLUT9). However, it facilitates electrogenic transport of both hexoses and uric acid in the proximal tubule (Witkowska et al., 2012). There are two forms, SLC2A9a and SLC2A9b, which are expressed in the basolateral and apical membranes of the proximal tubule, respectively. Their amino acid sequences are identical, except that SLC2A9b has a shorter and modified N-terminus. Both forms are active in urate transport in the proximal tubule (Kimura et al., 2014). Kidney function and SUA are interrelated (Kang et al., 2002). The anti-hypertensive drug losartan lowers SUA (Burnier et al., 1996; Wurzner et al., 2001; Hamada et al., 2002) and confers long-term protection of kidney function (Brenner et al., 2001). A recent GWAS conducted in Mexican Americans, reported a nominal association between UACR and *SLC2A9* SNPs (Voruganti et al., 2013). We found nominal associations between *SLC2A9* SNPs and kidney function phenotypes (Data not shown). Our results related to kidney function phenotypes replicate results of studies conducted in Mexican Americans and other American Indian tribes (Voruganti et al., 2013, 2014). However, our study is different from others as the participating individuals in our study were ascertained for CKD.

Total serum cholesterol was significantly associated with an intronic SNP (rs3852861) in the *PVRL2* gene on Chromosome 19. *PVRL2* is located 17 kb downstream from the apolipoprotein E (*APOE*) gene and has also been associated with severity of multiple sclerosis (Evangelou et al., 1999; Schmidt et al., 2002), late-onset Alzheimer’s disease (Corder et al., 1993), and peripheral T-cell lymphomas (Liestol et al., 2000). A study of Caucasian patients with coronary artery disease found a relationship between homozygosity of the A allele in a polymorphism of the *PVRL2/PRR2* gene and premature cardiovascular disease (Freitas et al., 2002). The authors suggested that this finding could be related to viral association or linkage disequilibrium between *PRR2* and nearby (17 kb centromeric) apolipoprotein E (*APOE*; Willer et al., 2008). This gene was also associated with LDL cholesterol in a Caucasian population although the chromosomal region is not the same (Talmud et al., 2009). We also found evidence of suggestive association of cholesterol esterase transfer protein (*CETP*) with HDL cholesterol which is a replication of several studies (Feitosa et al., 2014; Singaraja et al., 2014; Walia et al., 2014).

The association of triglycerides with four SNPs near and one SNP in the *BUD13/ZNF259* region replicates results observed in a Mexican cohort (Weissglas-Volkov et al., 2010), a meta-analysis of individuals of European descent (Schunkert et al., 2011), a Finnish cohort (Kristiansson et al., 2012) and Asian Indians (Braun et al., 2012). The minor allele frequency for rs964184 is higher among Zuni Indians (39%) than among Mexicans (27%), Asian Indians (15%) or Whites (12%). The *ZNF259/BUD13* associations with triglyceride levels have been reported in GWAS in the Framingham Study (Suchindran et al., 2010), which also showed an association with lipoprotein-associated phospholipase A2 (Lp-PLA2), a risk factor and possible therapeutic locus for CVD. Similarly, *ZNF259* was significantly associated with Lp-PLA2 activity in a meta-analysis of five population-based studies (Grallert et al., 2012). *ZNF259* codes for ZPR1, a zinc (as well as some other metals) binding protein, which may play a role in signal transfer from cell cytoplasm to the nucleus and cell proliferation (Galcheva-Gargova et al., 1998). This region of the *ZNF259/BUD13, APOA1/C3/A4/A5* genes has also been associated with coronary artery disease (Waterworth et al., 2010).

In addition, we also found some novel, albeit suggestive, associations of various SNPs with cardiometabolic phenotypes such as SNPs near *OLFML2B* and *NOS1AP* with blood pressure phenotypes. Although these SNPs have not been associated with blood pressure before, they have been associated with the Short QT-Syndrome among individuals from the UK and North America (Eijgelsheim et al., 2009; Nolte et al., 2009). Similarly, variants in the *NFIA* gene, which encodes the nuclear factor 1 family of transcription factors have been associated with QRS duration in individuals of European descent (Sotoodehnia et al., 2010).

### STRENGTHS AND LIMITATIONS

The strengths of our study include a dense GWAS using an Illumina chip that was state of the art at the time the study was conducted. Conducting the study in extended families from a relatively endogamous population increased our statistical power and minimized potential population stratification. Furthermore, we utilized state of the art programs for conducting genetic analyses (SOLAR, MERLIN). All study staff members working in Zuni were Zuni, which enhanced community acceptance of performing genetic studies in the Pueblo. Our study also had some significant limitations. We did not perform direct measurements of GFR. The serum creatinine assay was performed in a clinical laboratory and not standardized. The GFR estimating equation has not been validated in Zuni Indians. Kidney biopsies were not performed and may have led to misclassification. We did not account for all possible genetic X environmental interactions. In addition, we did not have positive controls. However, several of our significant loci have been previously identified in individuals without kidney disease.

## CONCLUSION

The results of the GKDZI study support our hypothesis that genetic factors significantly influence susceptibility to CKD and related cardiometabolic phenotypes among Zuni Indians.

## SUPPORT

This study was supported in part by grants DK066660-03 and DK57300-05 from the National Institutes of Health (NIH); 5M01RR00997 from the University of New Mexico Clinical Research Center; and P30 ES-012072 from the National Institute of Environmental Health Sciences, and Dialysis Clinic Inc. At the Texas Biomedical Research Institute, these studies were conducted in facilities constructed with support from the Research Facilities Improvement Program grant C06 RR013556 from the National Center for Research Resources, NIH. The AT&T Genomics Computing Center supercomputing facilities used for statistical genetic analyses were supported in part by a gift from the SBC Foundation. The statistical genetics computer package, SOLAR, is supported by grant R01 MH059490 from the National Institute of Mental Health.

## FINANCIAL DISCLOSURES

Philip G. Zager is an employee of both the University of New Mexico Health Sciences Center and Dialysis Clinic Inc. (DCI). Susan S. Paine is and Arlene Bobelu was a DCI employee. The remaining authors declare that they have no relevant financial interests. NIDDK appointed an independent Data Safety Monitoring Board, which had input into study design. Dialysis Clinic Inc., other than Philip G. Zager, Susan S. Paine, and Arlene Bobelu had no input into study design.

## Conflict of Interest Statement

Dr. Philip G. Zager is an employee of both the University of New Mexico and Dialysis Clinic Inc. The authors declare that the research was conducted in the absence of any commercial or financial relationships that could be construed as a potential conflict of interest.
